# Lymphangioma of the heart as a rare tumor: A case report

**DOI:** 10.1016/j.ijscr.2018.09.047

**Published:** 2018-10-08

**Authors:** Nádia Junqueira, Ricardo Ferreira, João Gonçalves, Ângelo Nobre

**Affiliations:** Cardiothoracic Surgery Department, Santa Maria Hospital, Lisbon, Portugal

**Keywords:** Case report, Lymphangiomas, Heart tumour, Rare tumour

## Abstract

**Introduction:**

Tumors of the heart are uncommon, and lymphangiomas are among the rarest of this group, with very few cases reported. These tumors consist of a benign slow-flow vascular malformation.

**Presentation of case:**

We report a case of a man diagnosed incidentally with a pericardial mass and our surgical approach for its treatment.

**Discussion:**

The pericardial mass of our case had no cleavage plane between the myocardium and the tumor. Because of this, rather than a total resection, with very high operative risk, a biopsy was preferred. The pathology specimen showed a benign tumor and the patient was referred to the cardiologist for regular follow-up.

**Conclusion:**

There is no specific investigation for diagnosing cardiac lymphangiomas, and though benign, they should be considered in the differential diagnosis of other malignant diseases of the mediastinum and definitive pathologic diagnosis is mandatory. Given the increased risk of uncontrolled bleeding related to percutaneous biopsy, the definite diagnosis is usually obtained with open surgical biopsy.

## Highlights

• Lymphangioma is a rare cardiac tumor.
• Lymphangiomas are benign in nature.
• Diagnosis based on imaging techniques is difficult.
• Surgery may be needed for definitive diagnosis and treatment.

## 1 Introduction

Tumors of the heart are uncommon and lymphangiomas are among the rarest of this group, with very few cases reported in the literature [1].

These tumors consist of a benign slow-flow vascular malformation containing lymphatic elements, forming a mass [2]. The head and neck are the most frequent locations for this tumors and they are more frequently observed during childhood. However, they may appear, or be detected, later in life as well [3].

Although cardiac lymphangioma is of benign nature, it should be considered in the differential diagnosis of vascularized soft tissue masses together with other more aggressive pathologies, such as angiossarcoma, lymphoma and metastatic disease. Usually, the diagnostic approach is surgical, given the increased risk of bleeding related to percutaneous biopsy [4]. Diagnosis based on imaging techniques is difficult, though the most useful method seems to be the magnetic resonance imaging (MRI) by elucidating the slow-flow components present in vascular malformations [5].

In line with the SCARE criteria, we report a case of a man diagnosed incidentally with a cardiac mass and our surgical approach [6].

## 2 Case report

**Fig. 1:**
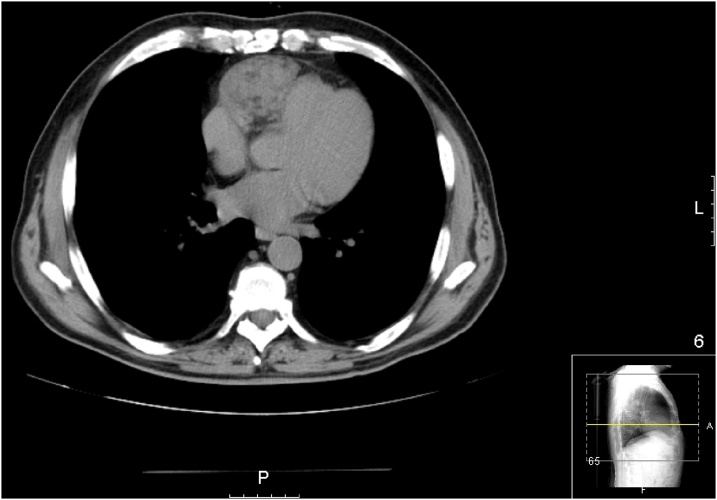
Computed tomography scan of the chest showing the right lateralized mass (asterisk), beginning in the antero-superior pericardium up to the right atrium (white narrow) and right ventricle (dotted arrow).

A 67-year-old male patient with no significant medical history presented to our institution to check a mediastinal enlargement incidentally found on routine chest X-ray. Echocardiographic examination revealed a solid mass surrounding the right cardiac chambers, and computed tomography of the chest confirmed the presence of a right lateralized 12 × 4 cm soft tissue mass beginning in the antero-superior pericardium recess down to the right atrium and right ventricle (Fig. 1). The mass did not contain calcification and it appeared adjacent with the right atrium. There was no pericardial effusion.

**Fig. 2:**
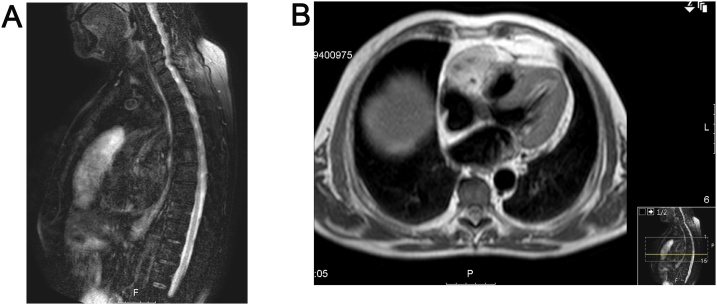
Magnetic resonance imaging in T2 showing the intrapericardial mass with hypersignal (asterisk) surrounding the wall of the right atrium (white narrow) and ventricle (dotted narrow) with apparent cleavage plan. **(A)** Sagittal section. **(B)** Axial section.

The subsequent techniques included a completely unremarkable coronariography with no signs of any neovascularization to the mass. The magnetic resonance imaging (MRI) confirmed the presence of an intrapericardial mass, with hypersignal in T2, localized in the anterior and superior pericardial recess, with inferior extension along the interatrial groove, and free wall of the right atrium and ventricle, surrounding the right coronary artery, but with apparent cleavage plane (Fig. 2).

**Fig. 3:**
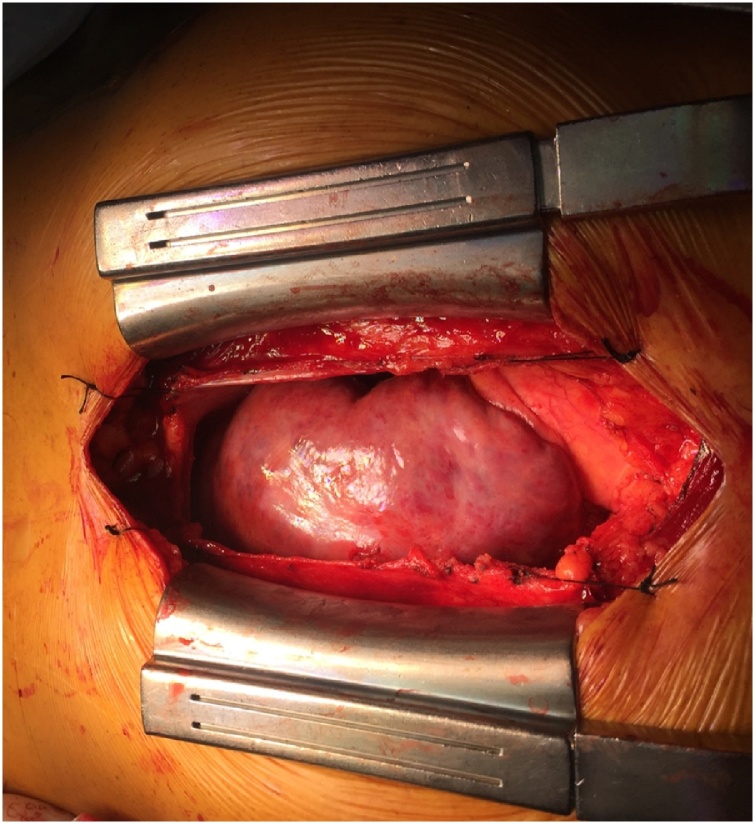
Tumor (asterisk) covering the surface of the right ventricle and right atrium. Aorta (black narrow).

**Fig. 4:**
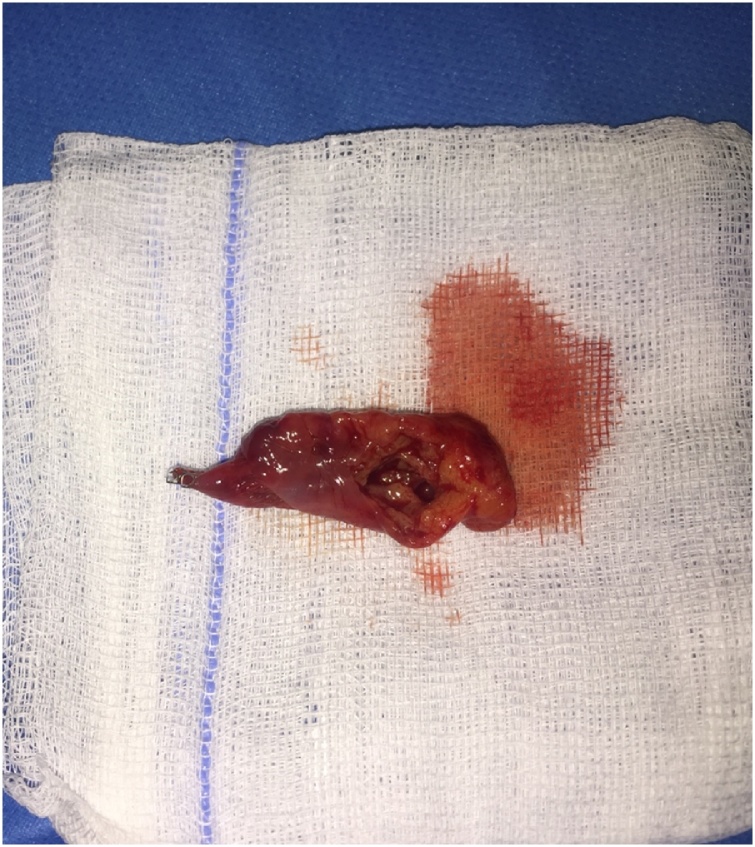
Macroscopic view of the biopsy taken from the tumor.

Median sternotomy approach was used to access the mass. The mass was completely adherent to the right atrium, right ventricle, and right coronary artery (Fig. 3). Due to this adherence, and the lack of a pathologic diagnosis, we ruled it unsafe to attempt a total resection of the mass, and instead performed a partial resection. The macroscopic examination of the cut surface revealed a large cystic space, with smaller spaces dispersed in a fibrotic wall (Fig. 4).

The patient had a normal post operative recovery and was discharged four days after the surgery. The patient was clinically well after one month.

The pathology specimen showed a mass containing lymphoid tissue, and the immunohistochemistry stains were consistent with a cardic lymphangioma.

## 3 Discussion

In this case, we have described a case of a pericardial mass for which the MRI suggested a cleavage plane between the myocardium and the tumor, which we were not able to confirm at surgery. Because of this, rather than a total resection with a very high operative risk, a biopsy was preferred due to the possibility of the tumor be benign.

The pathology specimen showed a benign tumor and at 1-month follow-up, the patient was clinically well. He was referred to the cardiologist for regular follow-up with serial echocardiograms to monitor the behavior of the mass.

## 4 Conclusion

Lymphangioma is a very rare vascular malformation, hence there are very few cases reported in the literature (Table 1). Consequently, no consistent guidelines for treatment and follow-up are available.

**Table 1:** Treatment options and outcomes of reported cases of cardiac lymphangioma.

STUDY	TREATMENT
*Nataf* *(1988)*	Complete ressection
*Nakazato* *(1995)*	Complete ressection by sternotomy
*Riquet* *(1997)*	Three cases Complete ressection by sternotomy
*Flörchinger* *(2005)*	Complete ressection by sternotomy under CPB and mitral valve reconstruction
*Pennec* *(2006)*	Surgical resection was impossible Cardioverter defibrillator was implanted
*Kim* *(2007)*	Complete ressection by sternotomy under CPB
*Kim* *(2010)*	Complete ressection by sternotomy under CPB
*Shroff* *(2011)*	Total resection
*Cailleba* *(2012)*	Total resection Partial resection of the right coronary artery
*Huang* *(2013)*	Complete ressection by sternotomy under CPB and cardiac arrest
*Biskupski* *(2013)*	Complete resection. Annuloplasty of the tricuspid valve was performed.
*Vinayakumar* *(2013)*	Surgical biopsy Pericardial window
*Robillard* *(2014)*	Surgical biopsy The mass has now been followed for >8 years with a minimal increase in size
*Lone* *(2016)*	Incomplete resection Follow-up for 2 years, showed no increasing in size
*Bansal* *(2017)*	Complete resection under CBP Skeletonizing the right coronary artery along the length of the mass

Search conducted in Pubmed using the keywords: lymphangioma, heart, cardiac tumour, CPB, cardiopulmonary bypass.

In our case, as the patient was entirely asymptomatic and complete resection would have been a very complex and dangerous procedure, we opted for the surgical biopsy. Even in cases when the MRI does not show a visible cleavage plane, the safest, and more suitable option, is always a surgical biopsy because these masses tend to bleed easily. Therefore, a differential diagnosis with consideration of other malignant diseases is necessary, since there is no specific investigation to identify this particular tumor.

## Conflicts of interest

The authors don’t have any financial and personal relationships that could influence their work.

## Funding source

Nothing to declare.

## Ethical approval

This is a case report. Informed consent was obtained from the patient and submitted to ethics committee of CHLN – Hospital Santa Maria according to protocol.

## Consent

Written consent was obtained from the patient for publication of this case and accompanying images. A copy of the written consent is available for review by the editor-in-chief of this journal on request.

## Authors contribution

**Ricardo Ferreira**, Study conception and design, data collection, revision of the paper.

**Nadia Junqueira**, Data collection and writing the paper.

**Joã**, Writing and revision.

**Angelo Nobre**, Revision and supervision.

## Registration of research studies

Not Applicable.

## Ethical Approval and consent participate

Manuscript was approved by the Ethics Committee of Hospital de Santa Maria and Centro Académico de Medicina de Lisboa. The reference number is 365/18.

A copy of the ethical approval is available for review by the editor-in-chief of this journal on request.

## Guarantor

Ricardo Ferreira.

## Provenance and peer review

Not commissioned, externally peer reviewed.
